# Differential effects of sex on hippocampal volume and cortical thickness in a racially and ethnically diverse cohort across the Alzheimer’s disease continuum

**DOI:** 10.1002/alz.087093

**Published:** 2025-01-09

**Authors:** Taylor F Levine, Jessica ZK Caldwell

**Affiliations:** ^1^ Cleveland Clinic Lou Ruvo Center for Brain Health, Las Vegas, NV USA

## Abstract

**Background:**

Although substantial literature supports sex differences in Alzheimer’s disease (AD), many research samples comprise predominantly non‐Hispanic white (NHW) participants, limiting generalizability. Work from our group in mainly‐NHW cohorts suggests a female‐specific time‐limited resilience in cortical thickness (CT; Cieri et al., 2022) and possibly hippocampal volume in the presence of amyloid (ADNI: Caldwell et al., 2017; Caldwell et al., 2018; but c.f., BioFINDER: Caldwell et al., 2019). Here, we investigated brain‐based sex differences in a racially and ethnically diverse AD cohort.

**Method:**

We obtained data from the Health and Aging Brain Study‐Health Disparities and included 798 self‐reported NHW, 470 Black American (BA), and 713 Mexican American (MA) participants. We examined sex differences in structural resilience, as defined by stable hippocampal volume (HCV) and cortical thickness (CT; including superior and middle temporal, isthmus and posterior cingulate, precuneus, and inferior parietal) in the presence of AD pathology (plasma Aβ_42_/Aβ_40_). We hypothesized that women would show structural resilience when cognitively normal (CN; CDR=0) but not when mildly cognitively impaired (MCI; CDR=.5‐1).

**Results:**

A significant moderation was observed for NHW individuals, such that CN women showed no relationship between HCV and Aβ_42_/Aβ_40_, whereas MCI women showed greater atrophy with increasing pathology (Right: p=.027; Left: p=.043). The same pattern was observed in BA men, who showed no relationship between HCV and Aβ_42_/Aβ_40_ when CN, but more atrophy with greater pathology when MCI (Right: p=.003; Left: p=.007). CT moderation analyses revealed a similar pattern of structural resilience in BA CN women in the left precuneus (p=.026) and left inferior parietal region (p=.026), and in MA CN men in right middle (p=.038) and left superior temporal regions (p=.028).

**Conclusion:**

As in some prior studies, CN NHW women showed time‐limited HCV resilience in the presence of amyloid; BA women also showed time‐limited CT resilience. Counter to expectations, resilience also was observed in CN BA men (HCV) and CN MA men (CT). Further work is needed to parse complex patterns of resilience and vulnerability across racial and ethnic groups, and to understand causal factors underlying these patterns.

